# New Insurance Plans

**Published:** 1920-03-20

**Authors:** 


### New Insurance Plans.

The *Times* Parliamentary correspondent indicates the higher rates of contribution which are embodied in the National Health Insurance Bill.

Generally speaking, the Government propose that the employer's contribution shall in future be 5d. a week, the male employed person's 5d., and the female employed person's 4d. The present rates of contribution are 7d. for men and 6d. for women, of which 3d. is paid by the employer and the remainder by the employed person. What the change will mean can be judged from the case of the domestic servant. At present her card has to bear a 6d. insurance stamp, of which 3d. is paid by the employer and 3d. by the employed person. In future her card will have to bear a 9d. stamp, of which 5d. will be paid by the employer and 4d. by the employed person. The State contribution will be, as at present, two-ninths of the combined contribution of the employer and the employed person in the case of a man and one-fourth in the case of a woman.

The general scope of medical benefit will not be changed by the Bill. It will, however, be followed by regulations which will lead to considerable modifications with a view to improving the standard of medical service and to simplifying its administration. Under the new regulations, a limit will be placed on the number of patients for whom a doctor can be responsible single-handed; a national maximum of 3,000 has been fixed. The scope of free choice of doctor has been enlarged; there will be a half-yearly choice instead of a yearly one. Express obligations are laid on doctors to give emergency treatment and to provide proper surgery and waiting-room accommodation.

